# Androgens in maternal vascular and placental function: implications for preeclampsia pathogenesis

**DOI:** 10.1530/REP-18-0278

**Published:** 2018-09-12

**Authors:** Sathish Kumar, Geoffrey H Gordon, David H Abbott, Jay S Mishra

**Affiliations:** 1 Department of Comparative Biosciences School of Veterinary Medicine, University of Wisconsin, Madison, Wisconsin, USA; 2 Department of Obstetrics and Gynecology School of Medicine and Public Health, University of Wisconsin, Madison, Wisconsin, USA; 3 Endocrinology-Reproductive Physiology Program University of Wisconsin, Madison, Wisconsin, USA; 4 Wisconsin National Primate Research Center University of Wisconsin, Madison, Wisconsin, USA

## Abstract

Adequate maternal vascular adaptations and blood supply to the uterus and placenta are crucial for optimal oxygen and nutrient transport to growing fetuses of eutherian mammals, including humans. Multiple factors contribute to hemodynamics and structuring of placental vasculature essential for term pregnancy with minimal complications. In women, failure to achieve or sustain favorable pregnancy progression is, not surprisingly, associated with high incidence of antenatal complications, including preeclampsia, a hypertensive disorder of pregnancy. While the pathogenesis of preeclampsia in women remains unknown, a role for androgens is emerging. The relationship between androgens and maternal cardiovascular and placental function deserves particular consideration because testosterone levels in the circulation of preeclamptic women are elevated approximately two- to three-fold and are positively correlated with vascular dysfunction. Preeclampsia is also associated with elevated placental androgen receptor (AR) gene expression. Studies in animal models mimicking the pattern and level of increase of adult female testosterone levels to those found in preeclamptic pregnancies, replicate key features of preeclampsia, including gestational hypertension, endothelial dysfunction, exaggerated vasoconstriction to angiotensin II, reduced spiral artery remodeling, placental hypoxia, decreased nutrient transport and fetal growth restriction. Taken together, these data strongly implicate AR-mediated testosterone action as an important pathway contributing to clinical manifestation of preeclampsia. This review critically addresses this hypothesis, taking into consideration both clinical and preclinical data.

## Introduction

Pregnancy is characterized by major cardiovascular adaptations, including marked decreases in systemic vascular resistance and mean arterial pressure, along with increases in maternal cardiac output and total blood volume (Magness 1998, Thornburg *et al.* 2000, Chinnathambi *et al.* 2013*a*). Studies suggest that pregnancy-enhanced vasodilatory actions allow peripheral vessels to accommodate increases in blood flow and volume (Conrad *et al.* 1993). Consistently, maternal vascular adaptations are accompanied by blunted vascular contractility (Naden & Rosenfeld 1981, Magness & Rosenfeld 1986) and enhanced release of endothelium-derived vasodilatory factors (Kawano & Mori 1983, Magness *et al.* 1990, 1996, 2000, Conrad *et al.* 1993, Sladek *et al.* 1997, Williams *et al.* 1997, Gillham *et al.* 2003, Gokina *et al.* 2010). Failure of these vascular adaptations during pregnancy are directly related to several maternal/fetal pathologies, such as increased systemic vascular resistance, hypertension, proteinuria, poor placental growth, decreased nutrient transport and low birth weight, all characteristics associated with a diagnosis of preeclampsia (Powe *et al.* 2011) outlined in Table 1. Despite being a leading contributor of maternal and perinatal morbidity and death worldwide, the etiology and pathogenesis of preeclampsia remain unclear (Roberts *et al.* 1991, Palei *et al.* 2013, Salam *et al.* 2015).

**Table 1:** Diagnostic criteria for preeclampsia in women (ACOG Guidelines 2013).

Parameter	Diagnostic criteria
Blood pressure	Greater than or equal to 140 mmHg systolic or greater than or equal to 90 mmHg diastolic on two occasions at least 4 h apart after 20 weeks of gestation in a woman with a previously normal blood pressure
	OR
	Greater than or equal to 160 mmHg systolic or greater than or equal to 110 mmHg diastolic, hypertension can be confirmed within a short interval (minutes) to facilitate timely antihypertensive therapy
Proteinuria	Greater than or equal to 300 mg per 24 h urine collection (or this amount extrapolated from a timed collection)
	OR
	Protein/creatinine ratio greater than or equal to 0.3*
	Dipstick reading of 1+ (used only if other quantitative methods not available)
OR in the absence of proteinuria, new-onset hypertension plus new onset of any of the following:	
Thrombocytopenia	Platelet count less than 100,000/microliter
Renal insufficiency	Serum creatinine concentrations greater than 1.1 mg/dL or a doubling of the serum creatinine concentration in the absence of other renal disease
Impaired liver function	Elevated blood concentrations of liver transaminases to twice normal concentration
Pulmonary edema	
Cerebral or visual symptoms	

*Each measured as mg/mL.

Treatment options for preeclampsia are limited to management of high blood pressure using antihypertensives, such as methyldopa, hydralazine, labetalol and nifedipine (ACOG 2013), as well as magnesium sulfate for prevention of eclamptic seizures (Al Khaja *et al.* 2014); however, these treatments have limited efficacy, and the only cure is the delivery of the placenta with baby, a totally undesired outcome before late preterm (≥34 weeks gestation). While the exact causes of preeclampsia remain unknown, a large body of evidence, supported by preclinical models of preeclampsia, indicates that abnormal placentation early in pregnancy is an important initial event in the onset of preeclampsia (Roberts & Redman 1993, Myatt 2002). Such preeclamptic abnormal placentation stimulates the production of anti-angiogenic factors and cytokines, resulting in generalized vascular dysfunction and the clinical manifestation of preeclampsia. In the past several years, dysregulation of steroid hormones, specifically increases in maternal testosterone levels, has emerged as an important endocrinopathy repeatedly associated with clinical manifestations of preeclampsia. In order to develop more effective therapeutic interventions for preeclampsia, it is important to fully understand the role of testosterone in maternal vascular and placental function, as well as blood pressure control, during normal pregnancy and in preeclampsia. This review critically addresses the hypothesis of testosterone-mediated pathogenesis of preeclampsia, taking into consideration data from both clinical (human) and preclinical (animal) studies.

## Testosterone levels in clinical preeclampsia

**Figure 1:**
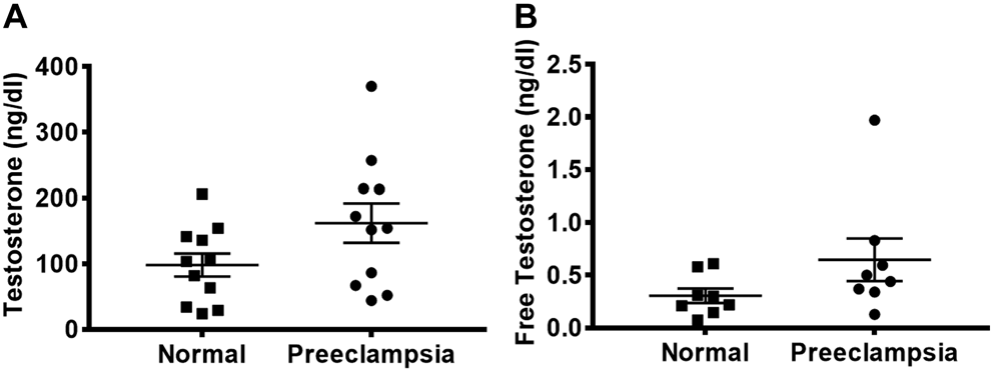
Mean higher total (A) and free testosterone (B) levels reported in preeclamptic patients compared normotensive controls in published studies cited in Table 1. Each point represents a single published study.

Most studies have investigated the beneficial role of sex steroid hormones, especially estradiol and progesterone, on cardiovascular function during pregnancy in women (Magness 1998). The relationship between testosterone and maternal cardiovascular function, however, is relatively understudied. PubMed search with keywords, ‘testosterone, preeclampsia and women’, generated 40 publications that were manually screened to identify 14 full-length papers reporting testosterone levels in both preeclampsia and control groups. Twelve of these 14 studies reported elevated plasma levels of testosterone during preeclamptic compared to normotensive (control) pregnancies (Table 2) (Acromite *et al.* 1999, Serin *et al.* 2001, Steier *et al.* 2002, Ficicioglu & Kutlu 2003, Miller *et al.* 2003, Troisi *et al.* 2003, Atamer *et al.* 2004, Baksu *et al.* 2004, Carlsen *et al.* 2005, Gerulewicz-Vannini *et al.* 2006, Salamalekis *et al.* 2006, Ghorashi & Sheikhvatan 2008, Hsu *et al.* 2009, Sharifzadeh *et al.* 2012). These studies report that during late pregnancy, plasma testosterone concentrations range between 100 and 150 ng/dL and these are 1.5- to 2.4-fold higher in preeclamptic compared to normotensive pregnant women (Fig. 1A). The reported mean unbound or ‘free’ testosterone level circulating in preeclamptic women is also 1.4- to 3.4-fold higher compared to normotensive pregnancies (Fig. 1B). Some studies also indicate that circulating testosterone levels correlate with the severity of preeclampsia, although this is not a universal finding (Ficicioglu & Kutlu 2003, Atamer *et al.* 2004). While there are many androgens, including the relatively bio-ineffective testosterone precursors of dehydroepiandrosterone (DHEA) and androstenedione (A_4_), only circulating levels of testosterone are increased during preeclampsia (Table 2). Preeclamptic hyperandrogenic measures include elevated total testosterone, free testosterone, free androgen index (FAI, total testosterone  × 100/sex hormone binding globulin) and the testosterone-to-estradiol ratio. Hyperandrogenic pregnant women with polycystic ovary syndrome (PCOS) are at increased risk for preeclampsia (de Vries *et al.* 1998, Kjerulff *et al.* 2011, Kamalanathan *et al.* 2013), and it has been proposed that overproduction of testosterone by the polycystic ovary is the causal factor engaging preeclampsia in PCOS women (Diamant *et al.* 1982, Sir-Petermann *et al.* 2002, Codner & Escobar-Morreale 2007). Obesity, and accompanying insulin resistance-induced compensatory hyperinsulinemia, is predictive of preeclampsia (Seely & Solomon 2003). Insulin stimulates androgen release, including testosterone, from theca cells of normal ovaries (Franks *et al.* 1999), and thus, the exaggerated hyperinsulinemia of obesity during preeclamptic gestation (Kaaja *et al.* 1995, Lorentzen *et al.* 1998) likely contributes to increased maternal testosterone levels (Andersen *et al.* 1995, Pasquali *et al.* 2000, Sutton-Tyrrell *et al.* 2010). Since both obesity, hyperinsulinemia and preeclampsia are more prevalent among hyperandrogenic pregnant women with PCOS than in pregnant women without PCOS (Lonnebotn *et al.* 2018), obesity-enhanced maternal testosterone levels may contribute to PCOS-associated preeclampsia.

**Table 2:** Plasma androgen levels in healthy and preeclamptic pregnant women.

Reference/Androgen	Normal vs preeclampsia	*P*	% Increase in preeclampsia^#^	Gestational age (weeks)
Acromite *et al.* (1999)				36–38
Total TS Free TS DHEA-S Estradiol	154.5 vs 213.6 ng/dL0.3 vs 0.5 ng/dL175.5 vs 171.0 µg/dL33.8 vs 36.4 g/mL	<0.01<0.05NSNS	3867	
Salamalekis *et al.* (2006)				30–31
Total TS Free TS DHEA-S Androstenedione	106.3 vs 154.4 ng/dL0.21 vs 0.34 ng/dL76.15 vs 57.62 µg/dL110.5 vs 107.1 ng/dL	<0.05<0.05>0.05>0.05	4562	
Ghorashi and Sheikhvatan (2008)				28–39
Free TS	0.58 vs 1.97 ng/dL	0.001	240	
Serin *et al.* (2001)				34–39
Total TS Free TS DHEA-S Androstenedione Estradiol	24.3 vs 44.1 ng/dL0.22 vs 0.44 ng/dL90.5 vs 162.5 µg/dL210 vs 220 ng/dL92.2 vs 73.5 pg/mL	<0.05<0.05NSNSNS	81100	
Carlsen *et al.* (2005)				33
Total TS Free TS index DHEA-S Androstenedione	63.4 vs 86.5 ng/dL0.61 vs 0.83102.3 vs 121.5 μg/dL280 vs 337 ng/dL	0.0010.012NSNS	3636	
Baksu *et al.* (2004)				34
Total TS Free TS index DHEA-S Estradiol	136 vs 257 ng/dL0.31 vs 0.37109.1 vs 104.3 µg/dL5830.1 vs 6164.2 pg/mL	0.0010.01NSNS	8919	
Steier *et al.* (2002)				30–38
Total TS	82.1 vs 172.4 ng/dL	<0.01	110	
Hsu *et al.* (2009)				37
Total TS	34 vs 52 ng/dL	<0.01	53	
Gerulewicz-Vannini *et al.* (2006)				37
Total TS Free TS DHEA-S	103.7 vs 152.2 ng/dL0.144 vs 0.594 ng/dL70.0 vs 51.1 µg/dL	0.020.002NS	47312	
Atamer *et al.* (2004)				34–35
Total TS DHEA-S Androstenedione Estradiol	29 vs 67 ng/dL108 vs 112 μg/dL189 vs 158 ng/dL2927 vs 3572 pg/mL	<0.001NSNSNS	131	
Troisi *et al.* (2003)				37
Total TS Androstenedione	141.9 vs 214.5 ng/dL316.0 vs 506.3 ng/dL	0.00070.004	5160	
Sharifzadeh *et al*. (2012)				32–33
Total TS Free TS DHEA-S	206 vs 370 ng/dL0.074 vs 0.12851 vs 75 μg/dL	<0.01<0.01NS	8073	
Miller *et al*. (2003)				35
Total TS Free TS index DHEA-S Estradiol	206 vs 147 ng/dL2.03 vs 1.5075 vs 75 μg/dL18,536 vs 9619 pg/mL	NSNSNSNS		
Ficicioglu and Kutlu (2003)				34–35
Total TS Free TS index DHEA-S Estradiol	218 vs 209 ng/dL4.16 vs 5.24104 vs 77 μg/dL21,880 vs 21,370 pg/mL	NSNS<0.05NS		

All these studies used immunoassays (ELISA and RIA) to measure TS levels. This raises concern regarding assay sensitivity and the specificity because of risk of cross-reactivity between steroids and their multiple placental metabolites. Recently, liquid chromatography tandem mass spectrometry (LC-MS/MS) has been suggested as the new ‘gold standard’ method for measurement of TS levels. This recommendation is more geared towards situations in which measurements of TS are below detectable levels (such as in hypogonadal men, women, children etc.) or in species for which no specific antibodies are available (such as sheep). Recent studies that compared the predictive values of TS levels measured by LC-MS/MS and immunoassay showed no significant difference between the two analytical methods (Czeloth *et al*. 2017, Mitchell 2012). The TS levels reported in the studies cited here may be appropriate for two reasons. First, the TS levels in pregnant women are within detectable range, and second, the objective is to detect relative change in preeclamptic group compared to controls.

# % increase is calculated as 100 x (preeclampsia - normal)/normal.

TS, testosterone; NS, not significant.

**Figure 2:**
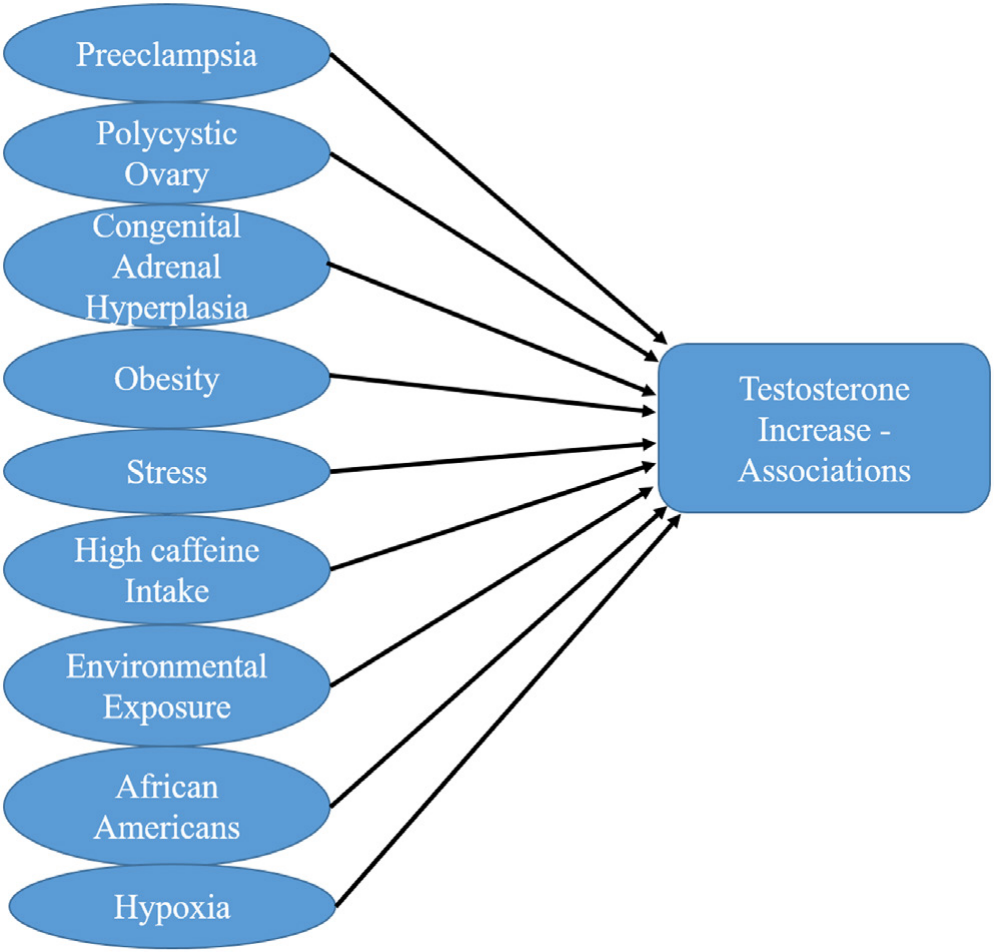
Possible associations for testosterone increase in females and pregnancy.

Ethnicity has also been implicated in contributing hyperandrogenism-related preeclampsia. Pregnant African-American women exhibit high maternal testosterone levels (120–130%), including elevated fetal cord blood testosterone levels at term (Henderson *et al.* 1988, Potischman *et al.* 2005, Rohrmann *et al.* 2009, Agurs-Collins *et al.* 2012) and are at increased risk for developing preeclampsia (Samadi *et al.* 2001, Iavazzo & Vitoratos 2010). In addition, plasma testosterone levels are increased during pregnancy in a variety of situations (Fig. 2), including classical congenital adrenal hyperplasia (Warmann *et al.* 2000, Mains *et al.* 2007), high caffeine intake (Ferrini & Barrett-Connor 1998, Svartberg *et al.* 2003) and stress (Sarkar *et al.* 2007, 2008), all of which are known risk factors for preeclampsia. Furthermore, pregnant women are inadvertently exposed to elevated testosterone levels via environmental pollutants and anabolic steroids (endocrine disruptors). High androgenic activity is reported in water from craft pulp and paper mills, as well as concentrated animal feed operations in the United States and Europe (Parks *et al.* 2001, Orlando *et al.* 2004). Reports have shown that an androgenic growth promoter used in beef cattle, trenbolone, has a half-life of greater than 260 days in animal by-products (Schiffer *et al.* 2001, Hotchkiss & Nelson 2007).

The degree of hyperandrogenism in preeclamptic women varies depending on the sex of their fetus. Preeclamptic women bearing sons exhibit higher testosterone levels than preeclamptic women bearing daughters (Steier *et al.* 2002), contributing to the notion that a male fetus and its placenta contribute additional amounts of testosterone to the maternal circulation (Sathishkumar *et al.* 2012). Such pregnant women bearing male fetuses are at increased risk for developing preeclampsia and placental dysfunction (Stark *et al.* 2006, Murji *et al.* 2012, Sykes *et al.* 2014, Li *et al.* 2018). Furthermore, daughters experiencing a preeclamptic gestation demonstrate higher circulating testosterone levels when they reach puberty (Alsnes *et al.* 2016). Such female offspring are at increased risk of developing hypertension and cardiovascular disease as adults (King *et al.* 2007, Sathishkumar *et al.* 2011*c*, Chinnathambi *et al.* 2012, 2013*b*, Vyas *et al.* 2016), and possibly preeclampsia and other pregnancy-related complications. High testosterone levels persist for at least 17 years in women with a documented history of preeclampsia (Laivuori *et al.* 1998). These studies thus provide consistent circumstantial evidence linking increased testosterone levels with preeclampsia.

**Figure 3:**
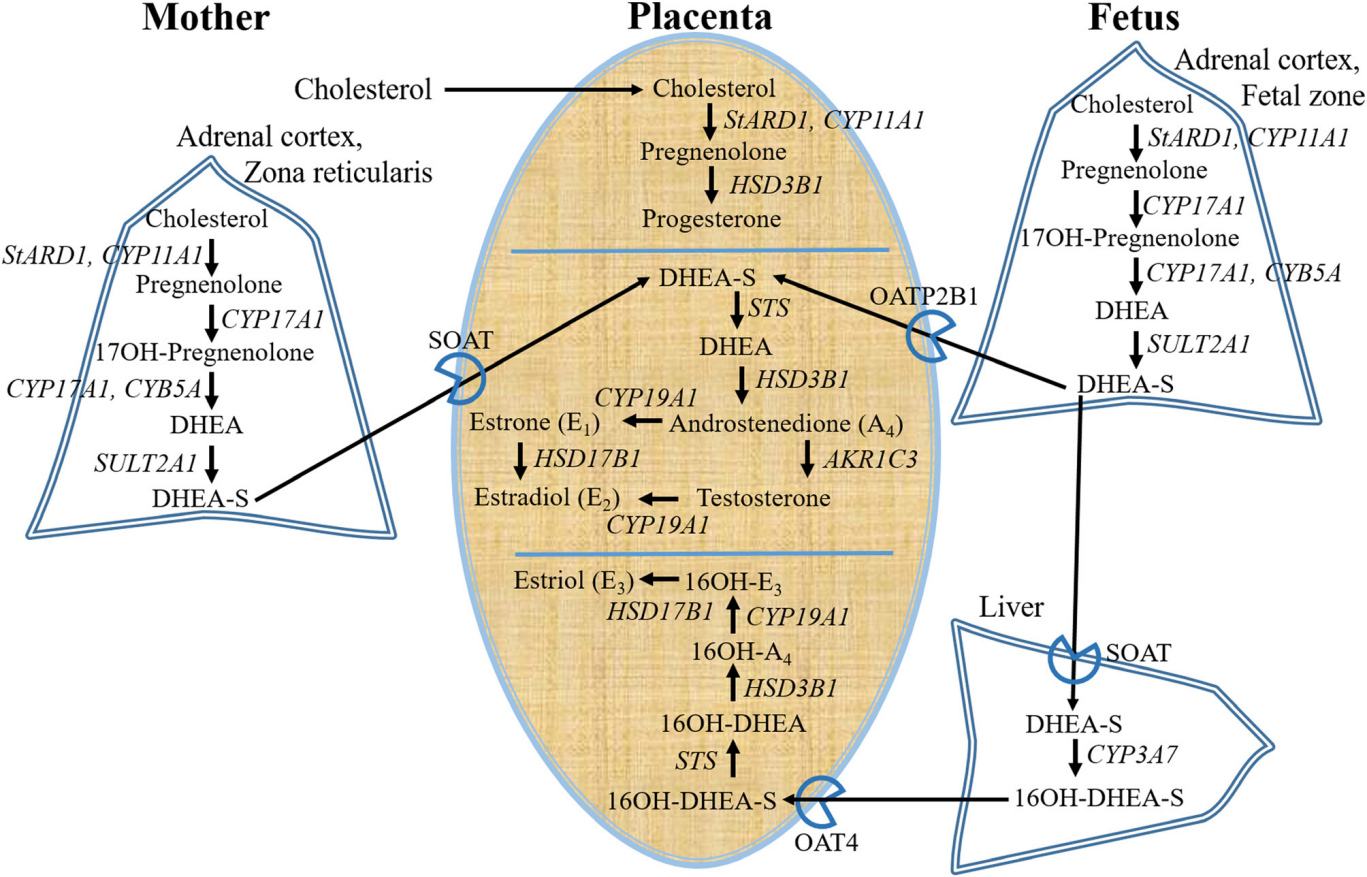
Pathway of biosynthesis and metabolism of testosterone, primary estrogens and progesterone during pregnancy. StARD1, steroidogenic acute regulatory protein; CYP11A1, cholesterol side-chain cleavage enzyme; HSD3B1, 3beta-hydroxysteroid dehydrogenase; CYP17A1, 17α-hydroxylase/17,20-lyase; CYB5A, cytochrome b5; SULT2A1, sulfotransferase; STS, steroid sulfatase; AKR1C3, aldo-keto reductase type 1C3; HSD17B1, hydroxysteroid 17-beta dehydrogenase 1; CYP19A1, aromatase – cell membrane-located uptake carriers of DHEAS; SOAT, sodium dependent organic anion transporter; OATP2B1 and OAT4, organic anion transporters.

The origin of the increased testosterone levels during preeclampsia remain uncertain. Studies suggest a placental contribution (Steier *et al.* 2002, Dokras *et al.* 2003). The human placenta, however, lacks the key androgen biosynthetic enzymes, 17β-hydroxylase and 17,20-desmolase (Christensen 1974). It nevertheless expresses 3β-hydroxysteroid dehydrogenase type 1 (HSD3B1) (Mason *et al.* 1993), endowing a ready ability to convert DHEA into A_4_, as well as the estrogen-preferring 17β-hydroxysteroid dehydrogenase type 1 (HSD17B1) (Takeyama *et al.* 1998), endowing a weak ability to synthesize testosterone from A_4_. After mid-gestation, both maternal and fetal adrenals equally contribute as the major sources of C19 steroids for placental androgen biosynthesis (Turnipseed *et al.* 1976, Kowalczyk *et al.* 1998). The human fetal adrenal cortex includes a fetal zone, expressing StARD1, CYP11A1, CYP17A1 and SULT2A1, essential for production of DHEA and DHEAS sulfate (DHEA-S), analogous to the maternal zona reticularis, the innermost zone of the maternal adrenal cortex (Fig. 3). After membrane uptake carrier transport into placental syncytiotrophoblast cells, sulfonated testosterone precursors (i.e. DHEA-S) are desulfonated by the enzyme sulfatase (STS) to yield DHEA. Placental steroidogenic enzymes (Fig. 3) then convert DHEA to A_4_ (HSD3B1), and A_4_ to testosterone (ARK1C3). Accompanying high levels of placental aromatase expression (Mason *et al.* 1993) ensure ready conversion of placental androgens, including testosterone, into non-androgenic, estrogenic metabolites (Gant *et al.* 1971, Buster *et al.* 1979, Dokras *et al.* 2003), including estrone, estradiol and their catechol and methoxy metabolites, some of which display placental bioactivity rivaling that of E_2_ (Jobe *et al.* 2011, Landeros *et al.* 2018).

The finding that there are no statistically significant differences regarding circulating maternal levels of DHEA-S and A_4_ between control and preeclamptic pregnancies implies that there is no contribution of adrenal steroids to the hyperandrogenism of preeclampsia. Placental aromatase mRNA and protein expression, however, is decreased in the preeclamptic placenta, diminishing metabolism of A_4_ and testosterone into estrogenic metabolites, and tipping the equilibrium between estrogens and androgens in favor of androgens (Sathishkumar *et al.* 2012, Perez-Sepulveda *et al.* 2015). Hepatic conjugation, and thus inactivation, of estrogens also appears diminished during preeclamptic gestations. Maternal circulating levels of unconjugated estrogens, however, remain unchanged from those in normotensive pregnant women (Rosing & Carlstrom 1984). Studies show that testosterone, alone, diminishes aromatase mRNA expression in human trophoblast cells through a miR-22-mediated mechanism (Shao *et al.* 2017). Other factors, tumor necrosis factor alpha (Lau *et al.* 2013) and lipid radicals (Mori *et al.* 2014), which are increased during preeclampsia downregulate aromatase (Milczarek *et al.* 2008, Diaz *et al.* 2009). In addition, hypoxia (which mirrors the actual conditions of the placenta in the context of preeclampsia) also downregulates placental aromatase (Jiang *et al.* 2000, Perez-Sepulveda *et al.* 2015, Yu *et al.* 2015). It would be interesting to assess whether compromised expression of aromatase also exists in tissues and organs other than placenta during preeclamptic gestation. A recent study also indicated that overexpression of CYP11A1 mRNA (commonly referred as cholesterol side-chain cleavage enzyme that catalyzes the first steps of steroidogenesis) in human trophoblast cells induces increased testosterone production and preeclampsia-like placental dysfunction that could be reversed with flutamide, an androgen receptor antagonist (Pan *et al.* 2017). Taken together, these findings support the notion that increased testosterone during preeclamptic pregnancies may be of placental origin, although other sources cannot be excluded.

## Testosterone and maternal blood pressure and uterine artery blood flow

In healthy women experiencing no complications during pregnancy, arterial pressure is stable during the early part of first trimester and then gradually decreases, reaching a nadir during the second trimester (Magness 1998, Bosio *et al.* 1999). Lack of this pregnancy-related decrease in blood pressure indicates a failure in normal cardiovascular adaptation and is considered to be a cardinal feature of preeclampsia (Ishikuro *et al.* 2012). Several independent investigators have demonstrated, through human and animal studies, the association of androgens, especially testosterone, with hypertension (Nakao *et al.* 1981, Reckelhoff *et al.* 1998, Gonzales *et al.* 2004, 2005, Park *et al.* 2004, Chen *et al.* 2007, Yanes *et al.* 2009, Makinen *et al.* 2011). Testosterone levels correlate positively with systolic blood pressure and diastolic blood pressure during and after preeclampsia (Laivuori *et al.* 1998, Serin *et al.* 2001, Carlsen & Heimstad 2012). Experimentally induced increases in maternal testosterone levels during pregnancy in rats, at concentrations that mimic testosterone levels found in human preeclamptic pregnancies, induce increases in systemic arterial pressure (Chinnathambi *et al.* 2013*a*, 2014*b*, Fornes *et al.* 2016), implying a causal role for testosterone in raising blood pressure during gestation. The exact mechanism by which testosterone mediates an increase in maternal blood pressure during pregnancy is not clear, but accumulating evidence indicates that testosterone increases vascular reactivity, activating the renin-angiotensin system and altering eicosanoid metabolism, thus favoring an increase in the thromboxane A2 to prostacyclin (PGI2) ratio and causing platelet aggregation in ways that are strikingly similar to those reported in preeclampsia (Acromite *et al.* 1999). Our unpublished observations also show that elevating rat maternal testosterone levels during pregnancy induce renal hypertrophy and proteinuria, a hallmark feature of preeclampsia (Sathishkumar *et al.* 2011*a*). Treatment with a selective angiotensin type 1 receptor (AT_1_R) antagonist, losartan, markedly attenuated the hypertension induced by testosterone in the pregnant rats (Chinnathambi *et al.* 2014*b*). These findings suggest that AT_1_R activation contributes, at least in part, to the testosterone-induced increase in blood pressure in rat pregnancies.

In addition to adjustments in systemic vasculature, the uteroplacental circulation normally adapts to maintain a low vascular tone to accommodate a more than 20-fold increase in uterine blood flow near-term (Rosenfeld *et al.* 1974, Magness 1998, Osol & Mandala 2009). Studies in hyperandrogenic women with PCOS have shown that their high maternal testosterone levels are associated with increased uterine artery resistance index and reduced blood flow (Palomba *et al.* 2010, 2012). Experimentally induced increase in maternal testosterone levels in pregnant rats show significantly reduced uterine arterial blood flow by 40% (measured using transcutaneous micro ultrasound) (Gopalakrishnan *et al.* 2016). In addition, elevated testosterone decreases uterine arterial diameter and increases resistance and pulsatile index (Gopalakrishnan *et al.* 2016). These findings suggest that the mechanisms controlling blood pressure and uterine artery hemodynamics during pregnancy are perturbed by elevated maternal testosterone levels. Primary estrogens, estrone, estradiol-17β and estriol play an important role in maintaining uterine blood flow and blunting vascular responses during pregnancy (Albrecht & Pepe 1990, Magness 1998). Jobe *et al.* (2013) elegantly demonstrated that these primary estrogens, and the majority of their catechol and methoxy metabolites, including those with demonstrable placental bioactivity (Jobe *et al.* 2011, Landeros *et al.* 2018), are reduced in preeclampsia (Jobe *et al.* 2013). The lower levels of primary estrogens, together with the reduced expression of placental aromatase could induce precursor steroid hormone accumulation, causing C19 steroids, especially testosterone, to be elevated. It is unclear if elevated testosterone acts independently or if it synergies with reduced downstream C18 estrogens to cause preeclampsia progression. Progesterone levels, however, are reported to be normal (Rosing & Carlstrom 1984, Bussen *et al.* 1998, Hertig *et al.* 2010), decreased (Acikgoz *et al.* 2013, Wan *et al.* 2018) or increased (Tamimi *et al.* 2003, Metz *et al.* 2014) in preeclampsia. Further studies will be needed to clarify whether there is hitherto unrecognized relationship between progesterone and testosterone, and if they work in concert in preeclampsia pathogenesis.

## Testosterone-induced mechanisms of vascular dysfunction during pregnancy

### Effects on endothelium-dependent relaxation

In humans, normal maternal vascular adaptations are accompanied by enhanced release of three major endothelium-derived vasodilatory factors including nitric oxide (NO) (Conrad *et al.* 1993, Sladek *et al.* 1997, Williams *et al.* 1997), PGI2 (Kawano & Mori 1983, Magness *et al.* 1990, 1996, 2000) and endothelium-derived hyperpolarizing factor (EDHF) (Gillham *et al.* 2003, Gokina *et al.* 2010). This is accompanied with concomitant pregnancy-induced increases in mRNA and protein expression of endothelial NO synthase (eNOS) (Sladek *et al.* 1997, Williams *et al.* 1997, Nelson *et al.* 2000, Magness *et al.* 2001), endothelial prostaglandin-I synthase (PGIS) (Bird *et al.* 2000, Magness *et al.* 2000) and EDHF activity (Gokina *et al.* 2010). In the systemic circulation, the principal endothelium-dependent vasodilators are NO and EDHF (Chinnathambi *et al.* 2013*a*). In the uterine arteries, in addition to NO and EDHF, PGI2 also plays a role in mediating vascular relaxation (Cooke & Davidge 2003). Elevated testosterone is shown to inhibit acetylcholine-induced relaxation of rat mesenteric and uterine arteries suggesting that elevated testosterone impairs endothelium-dependent relaxation. Specifically, the NO-mediated vasodilation was significantly decreased in mesenteric and uterine arteries in a pregnant rat model of elevated maternal testosterone (Chinnathambi *et al.* 2013*a*, 2014*a*). This testosterone-induced decrease in NO-mediated arterial relaxation was found not related to decreased vascular smooth muscle sensitivity to NO, as relaxation of arterial rings to sodium nitroprusside, an exogenous NO donor, was not affected (Chinnathambi *et al.* 2013*a*, 2014*a*). These findings indicate that testosterone likely alters synthesis/release of NO. Consistently, studies in rats have shown that testosterone decreases plasma levels of NO*_x_* (marker of NO production) with decreases in eNOS protein expression in uterine arteries (Chinnathambi *et al.* 2014*a*) and eNOS activity (decreased phosphorylation at excitatory Ser^1177^ site and increased phosphorylation at inhibitory Thr^495^ site) in mesenteric arteries (Chinnathambi *et al.* 2013*a*). The effect of testosterone in rat uterine arteries appears to be more profound than that in mesenteric arteries as in addition to decreasing NO pathway, it also compromises the EDHF- and PGI_2_-mediated relaxation by decreasing expression of small conductance calcium-activated channel-3 and PGI2 receptor, respectively (Chinnathambi *et al.* 2014*a*). Taken together, these results suggest that elevated testosterone during pregnancy may specifically impair the NO-mediated relaxation in systemic (mesenteric) vessels, while it compromises all three major vasodilatory pathways in reproductive (uterine) vessels.

### Effects on vascular smooth muscle contractile response

Systemic and uterine vasculature are refractory to vasoconstrictions during pregnancy. In contrast, enhanced contractile responses to vasoconstrictors is a characteristic feature of preeclampsia (Naden & Rosenfeld 1981, Magness & Rosenfeld 1986, Benoit *et al.* 2007, Stanhewicz *et al.* 2017). Elevated testosterone during pregnancy is shown to enhance contractile responses to many vasoconstrictors in endothelium-intact vessels, but in endothelium-denuded vessels, there is enhanced contractile response specific to angiotensin II in rat mesenteric (Chinnathambi *et al.* 2014*b*) and uterine arteries (Chinnathambi *et al.* 2014*a*). These enhanced responses observed in rat endothelium-denuded vessels indicate that enhanced arterial sensitivity is primarily because of increased angiotensin II-induced contractions, per se, rather than the loss of the endothelium-mediated relaxation component (Chinnathambi *et al.* 2014*a*). Since elevated testosterone does not alter the vasomotor response to other potent constrictors, such as K^+^ depolarization, thromboxane agonist U46619 and phenylephrine in endothelium-denuded vessels (Chinnathambi *et al.* 2014*a*,*b*), it appears that testosterone has a selective effect in enhancing vascular smooth muscle response to angiotensin II. It is possible that testosterone-mediated vascular smooth muscle dysfunction occurs at the agonist-specific receptor level rather than at common intracellular signaling pathways. Consistently, studies show that gestational elevation in testosterone levels causes selective upregulation of vasocontractile AT_1_ receptor and downregulation of vasodilatory AT_2_ receptor in mesenteric and uterine arteries implying that increased AT_1_/AT_2_ receptor ratio may play an underlying role in testosterone-induced exaggerated vasoconstriction to angiotensin II (Chinnathambi *et al.* 2014*a*,*b*).

## Testosterone on placental development and function

The progenitor cytotrophoblast cell is the stem cell of the placenta. These cells proliferate throughout gestation, differentiating along two pathways to form either villous cytotrophoblast, which ultimately can become syncytiotrophoblasts (outer cellular layer) or extravillous cytotrophoblasts (inner cellular layer). Syncytiotrophoblast is a specialized epithelium that has several functions, including transport of gases, nutrients and waste products and synthesis of peptide and steroid hormones that regulate placental, fetal and maternal systems. Extravillous trophoblasts have a proliferative component and an invasive component. There is also a migratory extravillous trophoblast, which is neither invasive nor proliferative. AR is present in syncytiotrophoblasts and in the decidua during the first trimester of human gestation (Horie *et al.* 1992). The expression of AR in human preeclamptic placentae is considerably higher than its expression in healthy placentae from uncomplicated pregnancies (Hsu *et al.* 2009, Sathishkumar *et al.* 2012). Also, genetic polymorphisms in the AR gene are associated with increased risk of preeclampsia (Lim *et al.* 2011). Rat models show that experimentally elevated maternal testosterone levels during pregnancy induce a reduction in placental size and weight (Sathishkumar *et al.* 2011*b*, Sun *et al.* 2012). The reason for smaller placenta in testosterone-exposed dams is not known, but may involve increased apoptosis or decreased proliferation (Ling *et al.* 2002). Pan *et al.* (2017) revealed a critical role for testosterone in human trophoblast invasion and demonstrated that flutamide, an AR antagonist, could rescue testosterone-induced reduction in invasion (Pan *et al.* 2017). It is possible that testosterone-induced autophagy (human) (Pan *et al.* 2017), reduced invasion (human) (Pan *et al.* 2017) or advanced placental differentiation (sheep) (Veiga-Lopez *et al.* 2011), may all contribute to such alterations in placental weight/morphology.

Vasculogenesis and angiogenesis are critical processes that lead to the formation of the placental vascular network necessary for optimal uteroplacental circulation (Huppertz & Peeters 2005, Arroyo & Winn 2008). Testosterone, however, downregulates the expression of genes related to vascular development and angiogenesis (*Ccr3*, *Stra6*, *Dhcr7*, *Arid1a*, *Ptprj*, *Col1a2*, *Lef1*, *Col1a1* and *Mmp2*) in the rat placenta (Gopalakrishnan *et al.* 2016). Along with this antivasculogenic gene expression profile, testosterone also decreases the radial and spiral artery diameters and inhibits branching angiogenesis (Gopalakrishnan *et al.* 2016). One of the important functions of the placenta is to promote nutrient transport to the fetus. Elevated testosterone is shown to decrease placental amino acid transport to rat fetuses (Sathishkumar *et al.* 2011*b*). This reduction in amino acid transport is related to reduced expression of the system A amino acid transporters (slc38a2/Snat 2) in the rat placenta (Sathishkumar *et al.* 2011*b*). Testosterone also decreases placental oxygenation with associated increase in hypoxia-inducible factor 1α and hypoxia responsive genes, presumable due to compromised placental vascularization (Gopalakrishnan *et al.* 2016). In addition to placental compromise, the fetuses of testosterone -exposed pregnant rats also receive less oxygen and are hypoxic (Gopalakrishnan *et al.* 2016). Elevated testosterone, however, does not alter glucose transport across the rat placenta (Sathishkumar *et al.* 2011*b*). Thus, testosterone increases during pregnancy alter placental structure and function leading to decreases in amino acid and oxygen availability to the fetus.

## Testosterone effects on fetal growth

Studies have shown that elevated maternal testosterone levels are associated with reduced birth weights in certain human populations (Sir-Petermann *et al.* 2005, Carlsen *et al.* 2006, Mehrabian & Kelishadi 2012), rats (Sun *et al.* 2012, Fornes *et al.* 2016), sheep (Manikkam *et al.* 2004, Steckler *et al.* 2005, Recabarren *et al.* 2008, Beckett *et al.* 2014) and marmoset monkeys (Smith *et al.* 2010), but not in rhesus monkeys (Abbott *et al.* 2010) and not in human populations of non-Spanish descent (Abbott *et al.* 2016). In rhesus monkeys, testosterone was experimentally increased during early-to-mid-gestation, 2 months prior to parturition, hence, it is possible that initial fetal growth restriction is masked by subsequent *in utero* catchup growth. Female infant monkeys, rats and sheep exposed to such gestational testosterone excess, however, exhibit accelerated body weight gain 2 months following parturition (Manikkam *et al.* 2004, Abbott *et al.* 2010, Sathishkumar *et al.* 2011*c*) and demonstrate increased abdominal adiposity and onset of type 2 diabetes and hypertension in adulthood (Chinnathambi *et al.* 2012, 2013*b*, Abbott *et al.* 2016). Testosterone is a lipophilic hormone and can diffuse across tissues, including placenta (Dell’Acqua *et al.* 1966, Meulenberg & Hofman 1991, Wang *et al.* 2005); however, whether fetal growth restriction induced by testosterone is the result of a direct effect on the fetus, or is secondary to decreased uterine blood flow or compromised placental function, remains to be resolved.

## Conclusions

**Figure 4:**
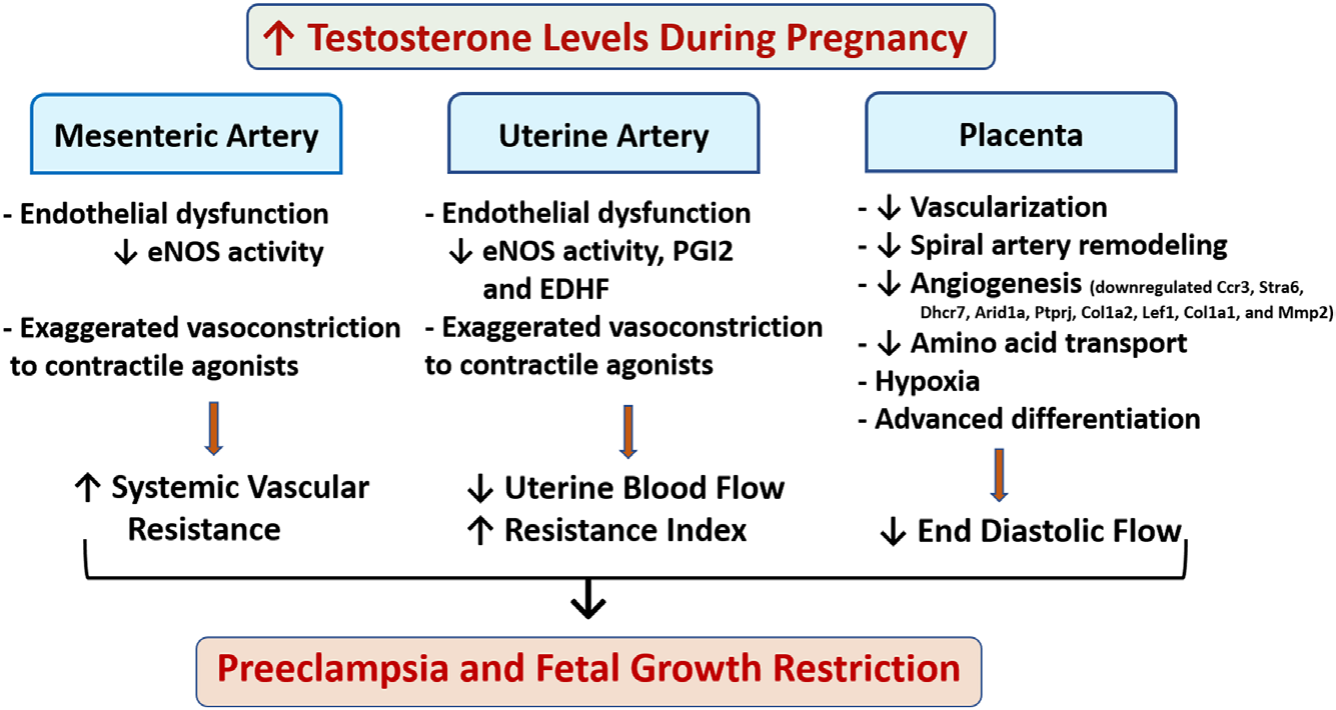
Unifying model depicting the central role of testosterone in preeclampsia. Increased testosterone level causes systemic, uterine and placental vascular dysfunction leading to increased blood pressure, decreased uterine artery blood flow and placental insufficiency, which may contribute to fetal growth restriction.

Several studies show that circulating levels of testosterone are two- to three-fold higher in preeclamptic pregnancies compared to those of healthy women experiencing uncomplicated pregnancies. Elevated testosterone in pregnant rats results in significantly increased arterial pressure and decreased uterine arterial hemodynamics. Testosterone, in pregnant rats, also causes endothelial dysfunction and exaggerated vasoconstriction to contractile agonists and dysregulates renin-angiotensin system with exaggerated vascular smooth muscle sensitivity to angiotensin II. In addition, testosterone compromises rat placenta vascularization and nutrient transport leading to placental hypoxia and fetal growth restriction. It is therefore possible that some of the vascular and placental effects observed during preeclampsia may indeed be testosterone mediated (Fig. 4). Therefore, strategies that (1) diminish excessive testosterone action in the cardiovascular and placental system and (2) identify the cause(s) of testosterone elevations during pregnancy, could have important therapeutic potential in treatment of pregnancies complicated by vascular dysfunction and fetal growth restriction.

## Declaration of interest

The authors declare that there is no conflict of interest that could be perceived as prejudicing the impartiality of this review.

## Funding

Financial support from the National Institute of Health (NIH) through grants HD069750, HL119869 and HL134779 (PI: S K), as well as HD044405 (PI: A Dunaif) and HD028934 (PI: J Marshall) supporting DHA, is greatly appreciated.
